# Examination of abiotic cofactor assembly in photosynthetic biomimetics: site-specific stereoselectivity in the conjugation of a ruthenium(II) tris(bipyridine) photosensitizer to a multi-heme protein

**DOI:** 10.1007/s11120-019-00697-8

**Published:** 2020-01-10

**Authors:** Nina S. Ponomarenko, Oleksandr Kokhan, Phani R. Pokkuluri, Karen L. Mulfort, David M. Tiede

**Affiliations:** 1 Chemical Sciences and Engineering Division, Argonne National Laboratory, 9700 South Cass Avenue, Argonne, IL 60439 USA; 2 Biosciences Division, Argonne National Laboratory, 9700 South Cass Avenue, Argonne, IL 60439 USA; 3 Department of Chemistry and Biochemistry, James Madison University, 901 Carrier Drive, Harrisonburg, VA 22807 USA

**Keywords:** Photosynthetic biomimetics, Circular dichroism spectroscopy, Stereoselectivity, Enantiomer, Ruthenium(II) tris(bipyridine), Multi-heme protein, Periplasmic cytochrome A

## Abstract

To understand design principles for assembling photosynthetic biohybrids that incorporate precisely-controlled sites for electron injection into redox enzyme cofactor arrays, we investigated the influence of chirality in assembly of the photosensitizer ruthenium(II)bis(2,2′-bipyridine)(4-bromomethyl-4′-methyl-2,2′-bipyridine), Ru(bpy)_2_(Br-bpy), when covalently conjugated to cysteine residues introduced by site-directed mutagenesis in the triheme periplasmic cytochrome A (PpcA) as a model biohybrid system. For two investigated conjugates that show ultrafast electron transfer, A23C-Ru and K29C-Ru, analysis by circular dichroism spectroscopy, CD, demonstrated site-specific chiral discrimination as a factor emerging from the close association between [Ru(bpy)_3_]^2+^ and heme cofactors. CD analysis showed the A23C-Ru and K29C-Ru conjugates to have distinct, but opposite, stereoselectivity for the *Λ* and *Δ*-Ru(bpy)_2_(Br-bpy) enantiomers, with enantiomeric excesses of 33.1% and 65.6%, respectively. In contrast, Ru(bpy)_2_(Br-bpy) conjugation to a protein site with high flexibility, represented by the E39C-Ru construct, exhibited a nearly negligible chiral selectivity, measured by an enantiomeric excess of 4.2% for the *Λ* enantiomer. Molecular dynamics simulations showed that site-specific stereoselectivity reflects steric constraints at the conjugating sites and that a high degree of chiral selectivity correlates to reduced structural disorder for [Ru(bpy)_3_]^2+^ in the linked assembly. This work identifies chiral discrimination as means to achieve site-specific, precise geometric positioning of introduced photosensitizers relative to the heme cofactors in manner that mimics the tuning of cofactors in photosynthesis.

**Electronic supplementary material:**

The online version of this article (10.1007/s11120-019-00697-8) contains supplementary material, which is available to authorized users.

## Introduction

Photosynthetic biohybrids created by the integration of synthetic photosensitizers within multi-cofactor redox proteins and enzymes are of growing interest because of the opportunities to use light-initiated single electron transfer chemistry to track intramolecular electron transfer pathways along cofactor arrays, and to follow intermediates during the charge-accumulating redox steps in hydrogen and nitrogen fixation reaction cycles (Brown et al. 2016; King 2018; Lam et al. 2016; Lee et al. 2018; Mulfort and Utschig 2016; Utschig et al. 2015). Additional opportunities have been demonstrated by coupling hydrogen-producing photosynthetic biohybrids to electron sources from photosystem II or water-splitting photoanodes, leading to the creation of complete, water-to-hydrogen, photosynthesis mimetic, Z-scheme hybrid architectures (Hutton et al. 2016; Kornienko et al. 2018; Utschig Lisa et al. 2018). A large range of photosensitizer motifs have been used in the design of photosynthetic biohybrids, including organic or inorganic molecular complexes, and visible-light absorbing nanoparticle or electrode semi-conductors (Lee et al. 2018). A research challenge now lies in achieving biohybrid designs with precise structural control of photosensitizer integration into the redox protein host assemblies, such that light-induced charge transfer and recombination dynamics can be optimized for photocatalytic function and precise control of the points of electron injection into multi-cofactor redox arrays.

Chirality in molecular complexes has been exploited as a means to confer conformational and site-specificity in photosensitizer labeling of proteins. For example, site-dependent stereoselectivity has been observed in the labeling redox proteins with ruthenium (II) polypyridine complexes and was applied to both heme proteins (Dmochowski et al. 2000; Luo et al. 1998) and proteolytic enzymes (Haquette et al. 2010). These studies demonstrate that complementary geometric shapes and non-covalent interactions within a protein binding pocket can be used to create a conformationally determined, lock-and-key specificity for photosensitizer integration within protein host matrices. Further, investigations of photosynthetic biohybrid designs for light-harvesting have demonstrated that chiral linkages can be used to create sterically-constrained, chromophore-protein couplings that function to enhance light-harvesting by modulating nuclear relaxation dynamics, extending excited-state lifetimes, and controlling Stokes shift energy losses (Delor et al. 2018). These designed protein-chromophore interactions are anticipated to mimic those functioning in native photosynthetic light-harvesting proteins.

In this report, we consider the effect of stereoselective conjugation of the photosensitizer, ruthenium(II)bis(2,2′-bipyridine)(4-bromomethyl-4′-methyl-2,2′-bipyridine), Ru(bpy)_2_(Br-bpy), to cysteine residues positioned by site-directed mutagenesis within the tri-heme cytochrome c_7_, PpcA from *Geobacter sulfurreducens.* PpcA is a small (10 kDa), robust, structurally and spectroscopically well-defined redox protein that makes it a useful platform to serve as a model for investigating mechanisms for photosynthetic biohybrid assembly. The protein sequence consists of only 71 amino acids and has one of the lowest amino acids to heme ratios among multi-heme cytochromes. The cofactors are c-type hemes, covalently bound in CXXCH amino acids motif and ligated by bis-histidine coordination. The crystal and solution structures of PpcA are known to high resolution (Morgado et al. 2012, 2017; Pokkuluri et al. 2004), and the redox (Morgado et al. 2010a), nuclear magnetic resonance (Morgado et al. 2010b), and electron paramagnetic resonance properties (Ponomarenko et al. 2018) of each of the hemes are distinguishable and well-characterized.

We have developed a series of photosensitizer-PpcA conjugates through the covalent linkage of Ru(bpy)_2_(Br-bpy) in a variety of positions along the PpcA polypeptide chain via cysteine introduced by genetic engineering (Kokhan et al. 2015). Photo-induced electron transfer (PET) times were found to vary from 6 × 10^−12^ to 4 × 10^−8^ s, correlated with the distance and pathways for electron transfer between photosensitizer and heme cofactors in these constructs (Kokhan et al. 2015). More recent work shows possibilities to create conjugates with even faster ET rates (Kokhan et al. 2017). Further, ultrafast PET has also been demonstrated in tetraheme heme protein architectures (van Wonderen et al. 2019). The cases of PET occurring on the few ps timescale are remarkable because these reactions begin to mimic the primary photosynthetic electron transfer steps and regarded as a goal for the design of biomimetic hybrids.

**Fig. 1:**
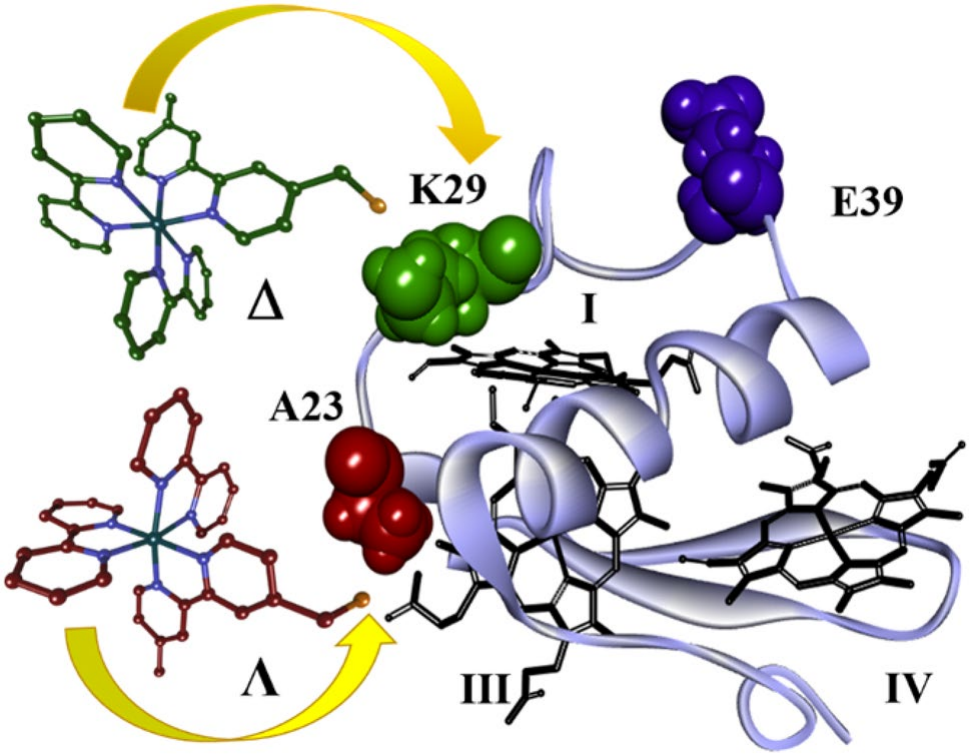
Structure of PpcA with location of amino acids replaced by cysteine for side-specific binding of the Ru(bpy)_2_(Br-bpy) photosensitizer, shown as Λ and Δ enantiomers directed to the position of preferred binding. The three heme groups are designated by Roman numerals in the order of attachment to the polypeptide chain and according nomenclature common with tetraheme c_3_ cytochromes

To gain insight into the linked structures underpinning the position-dependent PET rates, we investigated structural aspects of Ru(bpy)_2_(Br-bpy) conjugation by circular dichroism spectroscopy (CD) and molecular dynamics simulations (MD). The present investigation compares three conjugates, differing in sites for photosensitizer attachment and contrasting in rates of PET. As illustrated at Fig. 1, alanine A23 is positioned at the end of a short *α* helix and within van der Waals contact with one of the propionates for heme III. Lysine K29 is situated within the CXXCH binding domain for heme I but allows possibilities for the attached [Ru(bpy)_3_]^2+^ group to be in close vicinity to the vinyl groups for heme III. Glutamate E39 is located in a loop region almost equidistant from each of the three hemes (Pokkuluri et al. 2004). In addition to the interposition of the binding sites relative to heme cofactors, numbered by analogy to the structurally and evolutionarily-related tetraheme cytochrome c_3_ from genus *Desulfovibrio* (Matias et al. 1993), Fig. 1 shows the structures of the Ru(bpy)_2_(Br-bpy) *Λ* and *Δ* enantiomers. When these molecules are attached to the cytochrome, PET times for the K29C-Ru, A23C-Ru, and E39C-Ru conjugates are 6 ps, 130 ps, and 35 ns, respectively (Kokhan et al. 2015).

The CD and MD analysis of the A23C-Ru, K29C-Ru and E39C-Ru conjugates demonstrate a clear, site-dependent preference in the conjugation of a specific Ru(bpy)_2_(Br-bpy) enantiomer. The A23C-Ru and K29C-Ru conjugates show a distinct stereoselectivity for the opposite, *Λ* and *Δ*-Ru(bpy)_2_(Br-bpy) enantiomers, respectively, while the E39C-Ru has almost no selectivity. The degree of stereoselectivity was found to reflect the steric constraints and the precision in control of the [Ru(bpy)_3_]^2+^ conformation in the conjugated assembly. The spatial discrimination was identified as an element of the precise geometric positioning of introduced photosensitizer relative to the redox cofactors and the additional prerequisite of the fast electron transfer between photosensitizer and heme cofactor. In view of the growing applications of Ru(II) polypyridyl complexes as photosensitive groups (Brabec and Kasparkova 2018; Lam et al. 2016; Mital and Ziora 2018), the results depicted here are valuable for understanding their interactions with protein matrices and enhance our ability to design controlled electron transfer pathways in photo-activated biohybrids molecules.

## Methods

### Expression and purification of cytochrome

Recombinant expression was utilized to produce PpcA and cysteine mutants in *Escherichia coli* BL21. The heterologous expression system consisted of template plasmid pVA203 and accessory plasmid pEC86 bearing *ccm* genes essential for cytochrome c maturation (Thony-Meyer et al. 1995). The presence of accessory plasmid is required for heme synthesis, its subsequent transport through the cellular membrane and proper assembly of cytochromes in the periplasm. The Stratagene QuickChange II mutagenesis kit was used for introducing mutations into template pVA203. Purification of recombinant PpcA cytochromes was fulfilled by cation exchange chromatography according to (Londer et al. 2002). An additional treatment, involving the reduction of the engineered cysteine residues with 2 mM 1,4-dithiothreitol (DTT) for 30 min, was included at intermediate stage of purification. Size-exclusion chromatography using Superdex 75 column (GE Healthcare) equilibrated in 10 mM Tris–HCl buffer (pH 7.5), 100 mM NaCl was the final step in the purification of cytochromes bearing cysteine mutation.

### Binding photosensitizer molecule to cytochrome

Ru(II)(2,2′-bpy)_2_(4-(bromomethyl)-4′-methyl-2,2′-bpy)·2PF_6_, (where bpy corresponds to bipyridine), was synthesized following a previously published procedure (Gould et al. 1991). Binding of Ru(bpy)_2_(Br-bpy) to cysteine mutants of PpcA followed procedures described for functionalization of thiol-containing compounds in peptides, proteins and thiolated polynucleotides (Hansen and Winther 2009; Thiol-Reactive Probes 2010). To reduce cysteine before reaction, solutions of mutant PpcA (0.5–1 mM) were preincubated (room temperature, 30 min) with a fivefold molar excess of DTT, which was removed using a Sephadex G-25 column. The derivatization of cytochrome (0.1–0.5 mM) was performed at 4 °C in 10 mM Tris, pH 7.5, 100 mM NaCl buffer with a twofold molar excess of Ru(bpy)_2_(Br-bpy). After overnight gentle tumbling in a tube shielded from light, samples were centrifuged to remove residual aggregated proteins and insoluble Na[PF_6_]. The supernatant was concentrated using Amicon concentrators and applied to a Sephadex G-25 column combined with Superdex 75 column (GE Healthcare) to separate unreacted Ru(bpy)_2_(Br-bpy) from protein conjugate. During size-exclusion chromatography the elution progress was monitored as a function of time, with simultaneous UV–Vis absorption density measurements at three wavelengths: 286 nm for [Ru(bpy)_3_]^2+^ in the near-UV region; 406 nm for PpcA Soret band region; 529 nm peak for PpcA Q band region (SI Fig. S1). A clear separation of reddish Ru-PpcA band, having all three peaks of absorption, from an orange zone of non-reacted photosensitizer was evident during the chromatography process. The peak fractions of the first band, containing Ru-PpcA conjugate of each mutant, and the second, comprising the non-bound Ru(bpy)_2_(Br-bpy), were used for analysis by CD spectroscopy.

The covalent attachment of the photosensitizer molecule to the protein was verified with inductively coupled plasma mass spectroscopy (ICP-MS) on a Thermo Scientific iCAP 600 spectrometer. After determination Ru and Fe content, the Ru to PpcA ratio was calculated assuming 3 atoms of Fe per molecule of cytochrome.

Cytochrome concentrations in conjugates and parental mutants were evaluated spectrophotometrically using a molar absorption coefficient of 332.9 mM^−1^ cm^−1^ at 406 nm. In the determination of an extinction coefficient for PpcA at the Soret band absorption maximum, the quantitation of protein concentration was made using a Pierce 660 nm Protein Assay and verification of heme concentration was determined by Fe content measurement with ICP-MS.

Molar absorption coefficient values for estimation of [Ru(bpy)_3_]^2+^ concentrations have been taken from PhotochemCAD database (Taniguchi and Lindsey 2018).

### Circular dichroism spectroscopy

CD spectra were acquired on a JASCO J-810 spectropolarimeter in quartz 0.1 or 0.5 cm cuvettes at a scan speed of 50 nm min^−1^ using a 4 s average time, a 1 nm bandwidth, and a wavelength step size of 1 nm. CD spectra were measured as the difference between the spectra for each sample minus that of the corresponding buffer background, and each recorded as the average of 3 spectral scans. CD spectra in near-UV (230–360 nm) and visible (350–460 nm) spectral regions, corresponding to [Ru(bpy)_3_]^2+^ and heme groups, respectively, were obtained in 10 mM Tris buffer, pH 7.5 with 100 mM sodium chloride. To explore protein secondary structure in far UV (185–250 nm) region samples were transferred into 10 mM potassium phosphate buffer, pH 7.5, containing 30 mM ammonium sulfate, having lower absorption in this range. The concentration of cytochrome for different samples was in the range 18–33 uM.

The spectra presented as mean residue ellipticity [Θ]_mrw_, in deg cm^−2^ dmol^−1^, for analysis of protein secondary structure and molar circular dichroism *Δε*, in M^−1^ cm^−1^, for near-UV region according following equations:

1 $$\left[ \varTheta \right]_{\text{M}} = \, 100 \, \times \, \varTheta \, /M \, \times \, l$$

2 $$\left[ \varTheta \right]_{\text{mrw}} = \, 100 \, \times \, \theta /M \, \times \, l \, \times \, N$$

3 $$\Delta \varepsilon \, = \, \left( {\varepsilon_{\text{L}} {-} \, \varepsilon_{\text{R}} } \right) \, = \, \left[ \varTheta \right]_{\text{M}} / 3 2 9 8$$

4 $$\Delta \varepsilon \, = \, \left[ \varTheta \right]_{\text{mrw}} / 3 2 9 8,{\text{ for far UV CD of proteins}}$$

For these calculations Θ is the observed ellipticity in millidegrees, obtained during the acquisition of CD spectra, l is the optical path in cm, *M* is the molar concentration in mMol, *N* is the number of peptide bonds (Greenfield 2006; Kelly et al. 2005).

### Calculations of enantiomer ratio and enantiomeric excess

The identification of each of the enantiomers as either *Λ* or *Δ* was based on comparison of CD spectra to previously identified optical isomers of [Ru(bpy)_3_]^2+^ complexes. Before CD spectroscopic characterization enantiomers of these complexes were efficiently resolved by chiral chromatography (Browne et al. 2003; Caspar et al. 2003) or selective co-crystallization with a chiral compound (Chavarot et al. 2003; Hua and Lappin 1995; Noble and Peacock 1996), which ensured high levels of enantiomeric purity. The additional to CD analysis by MNR spectroscopy and X-ray crystallography (Browne et al. 2003; Caspar et al. 2003; Chavarot et al. 2003) provided higher level of confidence in determination of actual configuration of the complexes, and justified the use as the reference compounds.

The assignment of enantiomers is also supported by semi-empirical CD analysis rules, originating from the generalization of exciton chirality method and experimental data (Berova et al. 2007). The direction of the bisignate CD signal is determined by the relative chirality of the chromophore transition moments. For systems with positive chirality, a positive-to-negative sequence in the intensity of the peaks are observed with decreasing wavelength and inversion of this signal is seen for systems of opposite chirality (Pescitelli et al. 2014).

For the quantitative representation of enantiomeric composition, we calculated enantiomeric excess (ee), which has been defined as the excess of one enantiomer (E1 or E2) over the racemic composition in the mixture E1 + E2 (Schurig 2013). For chiroptical methods, CD, in particular, ee determination is based on optical purity (op) (Lopes et al.), which is the ratio of the observed specific rotation [*α*] of an enantiomeric mixture, divided by the maximum specific rotation [*α*_max_] of one enantiomer (E1 or E2 with ee = 1) at corresponding wavelength:

5 $${\text{op }} = \, \left[ \alpha \right]/\left[ {\alpha_{ \hbox{max} } } \right] \, = \, \left( {E1 - E2} \right)/\left( {E1 + E2} \right) \, = {\text{ ee}}$$

For calculation of the enantiomeric excess we used the *Δε* of each conjugate determined at 295 nm as the specific rotation value. As maximum specific rotation used *Δε*_295_ = + 114 M^−1^ cm^−1^ taken from the reference compound cis-*Λ*-[Ru(bpy)_2_(py)_2_]Cl_2_ presented in (Hua and Lappin 1995).

The enantiomeric proportions, E1 and E2 mole fractions and enantiomeric ratio (er), were determined from op and ee according to (Carey and Sundberg 2007; Schurig 2013)

6 $$E 1 { } = \, \left( { 1 { } + {\text{ op}}} \right)/ 2 {\text{ and }}E 2 { } = \, \left( { 1 { } - {\text{ op}}} \right)/ 2$$

7 $${\text{er }} = \, E_{\text{major}} /E_{\text{minor}}$$

We also used selection ratio coefficient characterizing domination of delta over lambda enantiomer, *Δ*/*Λ*, to explicate comparison of the three examined constructs.

### Molecular dynamics simulations

All-atom molecular dynamics (MD) simulations were performed using the Argonne Leadership Computing Facility (ALCF). In our MD simulations, the two model systems consist of a modified PpcA cytochrome in which the amino acid residue at position 23 or 29 is replaced by cysteine with covalently attached [Ru(bpy)_3_]^2+^. The molecular structure and atom numbering of the covalently attached [Ru(bpy)_3_]^2+^ molecule is shown in Fig. S2 of Supplemental Information. The initial atom coordinates of PpcA were taken from the solution structure deposited by Morgado and co-workers (Morgado et al. 2012), Protein Data Bank entry: 2LDO. In silico mutations were introduced using the VMD program (Humphrey et al. 1996). All MD simulations were performed using the NAMD2 package (Phillips et al. 2005). The systems with an explicit water box were allowed to equilibrate in all-atom MD simulations for ~ 400 ns with the following simulation settings: the constant temperature of 310 K and the constant pressure of 1 atm, 1 fs integration time, 12 Å cutoff distance. The electrostatic forces were calculated every four steps and nonbonded forces were calculated every two steps using the particle-mesh Ewald method. Both the temperature and pressure of the system were controlled with Langevin piston and bath as implemented in NAMD2. Force field parameters for Ru(bpy)_2_(Cys-bpy) were developed following the standard guidelines for CHARMM force field (Brooks et al. 2009; MacKerell et al. 1998), for c-type heme in cytochrome were taken from Autenrieth et al. (Autenrieth et al. 2004) with modified charge distribution resultant from bis-His axial ligation. For both A23C-Ru and K29C-Ru conjugates, three independent simulations were performed. The coordinate trajectories were recorded every 10 ps, analyzed and visualized using VMD 1.9.3 package (Humphrey et al. 1996).

## Results

### Absorption spectra

**Fig. 2:**
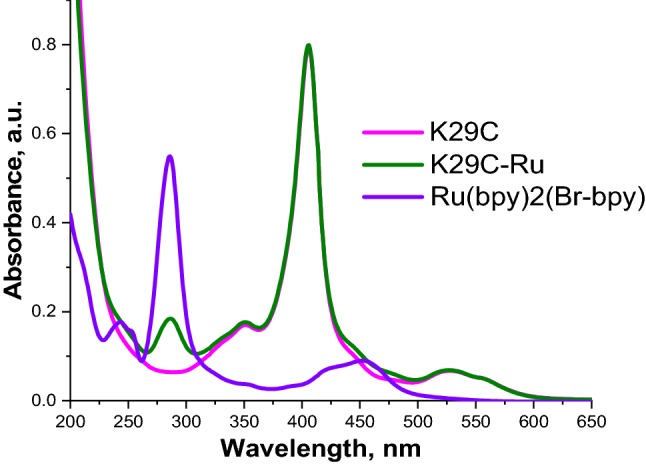
Optical absorption spectra of the PpcA mutants and conjugates, represented by K29C mutant (magenta), the linked K29C-Ru conjugate (olive) and Ru(bpy)_2_(Br-bpy) (violet). The K29C and K29C-Ru spectra recorded in 10 mM Tris, pH 7.5, 100 mM NaCl buffer, Ru(bpy)2(Br-bpy)—in acetonitrile

Conjugation of [Ru(bpy) _3_]^2+^ photosensitizer molecule to the protein scaffold is evidenced by the emergence of its absorption bands integrated with PpcA cytochrome spectrum in chromatographically purified samples. For example, Fig. 2 shows the optical absorption for the K29C-Ru assembly, compared to the K29C and Ru(bpy)_2_(Br-bpy) starting materials. The major peaks for the photosensitizer are seen in the conjugate, the ligand-centered *π*–π* transition (LCT) is located in the near-UV region at about 286 nm, and the metal-to-ligand transition (MLCT) in visible region is discernible as the shoulder on the high energy side of the heme’s Soret band around 457 nm.

### The far UV CD region (185–250 nm), secondary structure analyses of PpcA-Ru conjugates

**Fig. 3:**
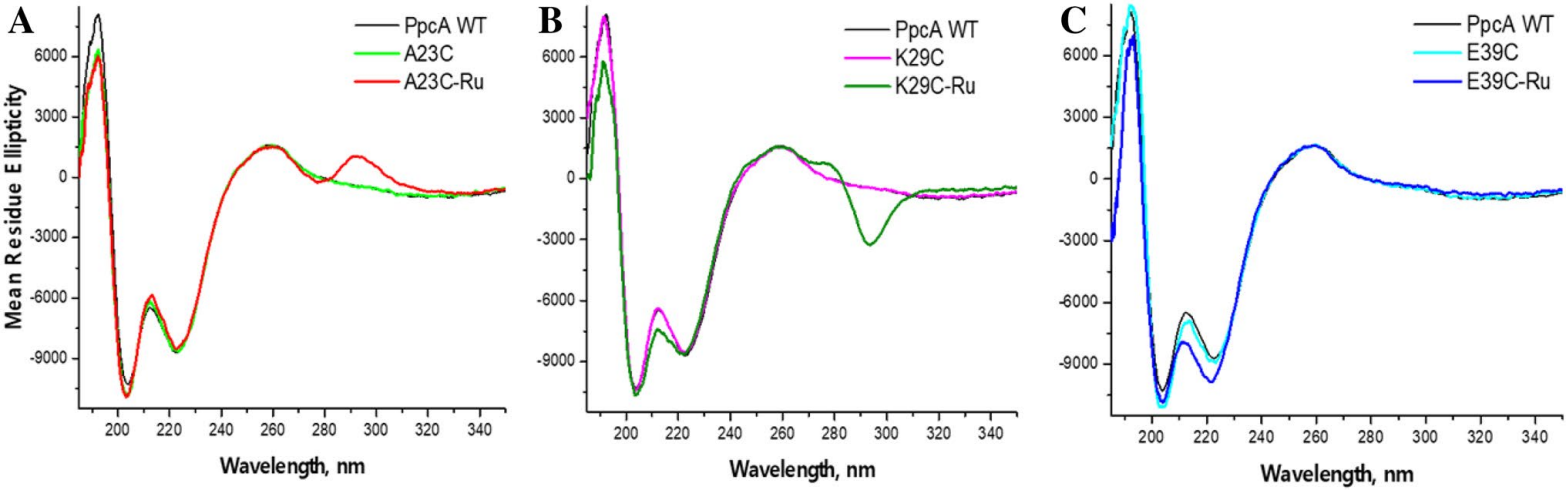
Circular dichroism spectra of PpcA-Ru conjugates in far UV region in comparison with corresponding mutants and wild-type cytochrome. a A23C-Ru, b K29C-Ru, c E39C-Ru. Experimental conditions: concentration of cytochrome moiety 18–21 uM, pathlength 0.1 cm, 10 mM potassium phosphate buffer (pH 7.5) containing 30 mM (NH_4_)_2_SO_4_. The spectra presented as mean residue ellipticity [Θ]_mrw_, in deg cm^−2^ dMol^−1^, the value removing the linear dependence from pathlength, protein concentration, length of the polypeptide chain and allowing the comparative analysis of protein secondary structure

In the far UV spectral region associated with optical absorption from the peptide bond, the CD spectra of PpcA and variants with Cys mutation introduced in 23, 29 and 39 positions are dominated by two negative minima and positive maximum near 190 nm (Fig. 3). The positions of bands are consistent with Cotton effects observed for helical polypeptide chains, where negative minima around 222 and 208 nm correspond to *n*–π*and *π*–π* ‖ transitions, maximum around 190 nm to *π*–π* ***⊥*** transitions of the amide bonds in a protein backbone (Bulheller et al. 2007; Greenfield 2006; Kelly et al. 2005). At the same time, the overall shape of the spectrum indicates the presence of *β*-conformation in combination with unordered elements since the lower wavelength band is found at 204 nm instead of typical for *α*-helix 208 nm, as detailed in the subsequent description.

For the quantitative characterization of conformational changes induced by cysteine mutations and then binding of Ru(bpy)_2_(Br-bpy) photosensitizer, we analyzed the helical content of polypeptide chains based on the ratios of ellipticities at extrema of CD spectrum, *R*_1_ ≅ [Θ]_max_/[Θ]_min_ and *R*_2_ ≅ [Θ]_222_/[Θ]_min_, Table 1. These indexes are useful as probes for the relative amounts of helical elements in similar peptides, thus the deviations in the ratios are sensitive indicators of the conformational disposition in a peptide backbone (Bruch et al. 1991; Manning and Woody 1991). The index *R*_1_ was found to be the most informative for helical content characterization because of the common origin of the two bands, due to exciton splitting of the amide *π*–π* transition in a helical polypeptide (Banerjee and Sheet 2017). The index *R*_2_, corresponding to the ratio of *n*–π* and *π*–π* ‖ transitions in peptide bonds, provides a characterization of the type of helices contained in a protein, as theoretical calculations (Manning and Woody 1991) suggest that both 3_10_- and *α*-helices may display similar 222-nm bands but 3_10_-helices should have more intense 208-nm bands. For peptides with low *α*-helices and high 3_10_-helices content *R*_2_ index anticipated to be lower than 0.9. In PpcA *R*_2_ = 0.85 because, as known from the crystal structure, this protein comprises only a few small *α*-helices along with a 3_10_-helix in holding heme I segment (Pokkuluri et al. 2004). Introduction of cysteine mutations had almost no influence on the main *α*-helix band at 222 nm, its position or intensity as apparent from Fig. 3. The *π*–π* ‖ transitions from amide bonds are to some extent altered, as evident by more negative minimum around 204 nm, especially in A23C and E39C mutants. The [Θ]_222_:[Θ]_204_ ratio decreases slightly from 0.85 for the native protein to 0.82 for K29C and 0.80 in A23C and E39C. In addition, for the A23C mutant, the intensity of 190 nm band also diminished, lowering the *R*_1_ index as well, to 0.60 from 0.79 in wild-type PpcA. Thus, the introduction of a Cys mutation at this site had the largest impact on secondary structure. This change is conditioned by the increase in size, polarity and hydrophobicity of Cys relative to replaced Ala, located, according to the crystal and solution structures of PpcA (Morgado et al. 2012; Pokkuluri et al. 2004), in proximity to propionate side chain attached to C-13 of heme III. This was the only Cys mutation where the substituted amino acid was smaller than the introduced one, thus some rearrangement in the surrounding area was required to accommodate the increased volume of introduced Cys residue, resulting in the biggest change detected by CD. For the other two mutants discussed here, K29C and E39C, the introduced Cys side chain was smaller than those of the original amino acids, what simplified its spatial accommodation. We assume that the loss of charges, positive in the K29C and negative in E39C, with accompanying interactions were the main reasons for the detected modifications in these two mutants.

**Table 1:** Helicity of cysteine mutants and constructs

	[Θ], 192 nm	[Θ], 204 nm	[Θ], 222 nm	R_1_, [Θ]_192_/[Θ]_204_	R_2_, [Θ]_222_/[Θ]_204_
PpcAWT	8105.07	− 10289.26	− 8724.04	0.79	0.85
A23C	6422.97	− 10,778	− 8588.90	0.60	0.80
A23Ru	5968.96	− 10942.95	− 8525.39	0.55	0.78
K29C	7954.39	− 10,451	− 8561.98	0.76	0.82
K29Ru	5715.61	− 10680.08	− 8626.80	0.54	0.81
E39C	8487.17	− 11108.74	− 8931.36	0.76	0.80
E39Ru	6905.94	− 10820.73	− 9876.29	0.64	0.91

Reaction of Ru(bpy)_2_(Br-bpy) with the A23C thiol resulted in a methyl bridged attachment of [Ru(bpy)_3_]^2+^, and induced a change in the *R*_2_ ratio of the negative UV CD peaks further, from 0.80 to 0.78, but the change in this index, and *R*_1_ as well, is smaller than just after the introduction of the Cys mutation. Conjugation of [Ru(bpy)_3_]^2+^ to K29C mostly resulted in a change of *R*_1_ index due to the decrease of the intensity of 190 nm peak. The *R*_2_ ratio remained close to the unlabeled form, but the shape of the spectrum was altered, and an additional element is noticeable between the peaks, at 211–217 nm, possibly resulting from a slight increase in *β* structure elements at the expense of helical content. In the E39C-Ru conjugate, the similar change in the shape of the spectrum between negative peaks was observed along with more negative ellipticity in this region, presumably indicating the small increase in secondary structure elements.

In essence, the far UV spectral region of PpcA, its cysteine mutants and conjugates as well, are characteristic of an *α* + *β* protein class having *α* helices and *β* sheets in relatively separate domains along the polypeptide chain (Manavalan and Johnson 1983; Venyaminov and Yang 1996). At the same time, the spectra display some distinctive features differing them from a typical *α* helix UV CD patterns, such as the position of the lowest wavelength minimum, which is found at 204 nm instead of 208 nm and its more prominent extremum than the 222 nm band. These details likely reflect the significant content of very short irregular strands classified as unordered secondary structures or “random coil” elements which have a UV CD minimum at ~ 200 nm (Lopes et al. 2014; Sreerama and Woody 2004). PpcA has the globular protein folding motif shared among the c_3_ and c_7_ cytochromes, characterized by a relatively small content of *α* helix and *β* sheet secondary structure elements which are connected by loops and unordered peptide segments (Morgado et al. 2012, 2017; Pokkuluri et al. 2004). This distribution of protein structural elements is understood to be reflected in the UV CD spectrum, position and interrelation of minima for PpcA as well as for the photosensitizer conjugates. In addition, protein UV CD spectra showing negative band features near 200 nm have been detected in proteins having extensive loop regions or irregular structures, along with disulfide bonds, for example, bovine pancreatic trypsin inhibitor or elastase represented in Protein CD Data Bank (Lees et al. 2006; Whitmore et al. 2017). Thus, the covalent binding of heme cofactors through thioether bonds in PpcA could be the additional factor influencing the shape of PpcA CD spectrum.

Overall, the UV CD spectra of [Ru(bpy)_3_]^2+^-linked constructs display only moderate alterations comparative to the wild-type PpcA, suggesting that there are no major rearrangements in the global secondary structure upon covalent linkage of the supplementary molecule. Presumably, the detected relatively small changes reflect the local adjustments of side chains of amino acids required for spatial accommodation of the synthetic photosensitizer cofactor.

### CD spectra in the near-UV region (230–360 nm), coordination and enantiomer selectivity in Ru(bpy)_2_(Br-bpy) photosensitizer ligation

**Fig. 4:**
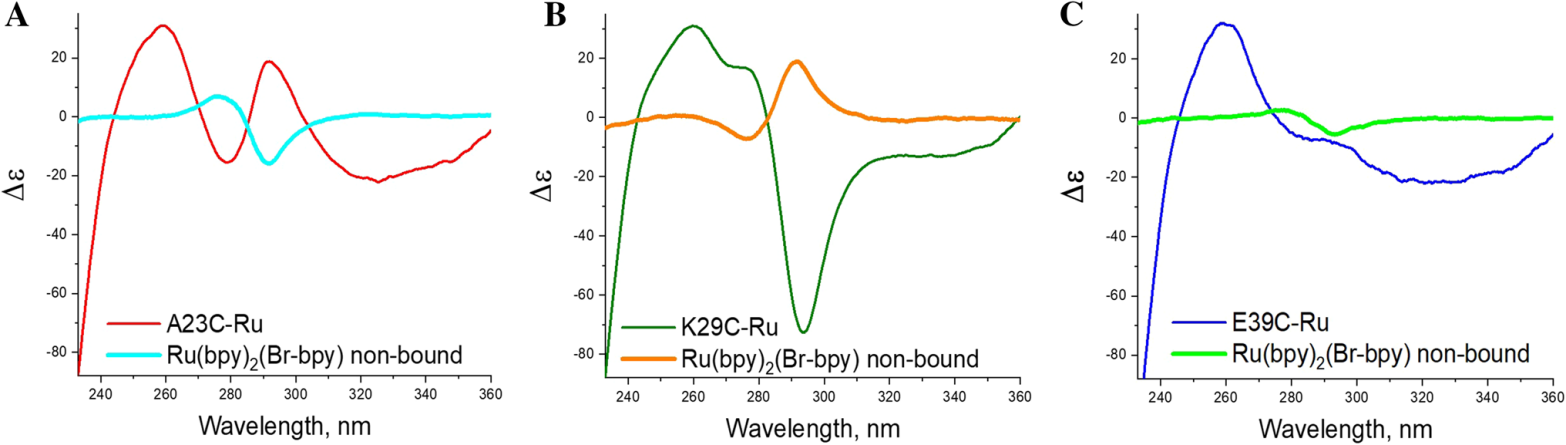
CD spectra of Ru-PpcA constructs and non-bound Ru(bpy)_2_(Br-bpy) separated by size-exclusion chromatography after linking reaction. a A23C-Ru; b K29C-Ru; c E39C-Ru. Experimental conditions: estimated concentration of cytochrome 20.6–32.8 uM, Ru(bpy)_2_(Br-bpy) 86–193 uM, pathlength 0.5 cm, 10 mM Tris buffer, 100 mM NaCl. The spectra presented as molar circular dichroism Δε, in M^−1^ cm^−1^

The near-UV CD spectra for the PpcA-Ru constructs in the region of the [Ru(bpy)_3_]^2+^ LCT band, show diametrically opposed Cotton effects in two of the investigated variants. A23C-Ru exhibits a positive peak (at 293 nm), while K29C-Ru has a larger, negative one (at 294 nm), Figs. 3 and 4. The E39C-Ru construct shows a very mild positive effect. The observed bisignate couplets are analogous to spectra for the *Λ* and *Δ* enantiomers of [Ru(bpy)_3_]^2+^ and its derivatives described in (Browne et al. 2003; Caspar et al. 2003; Chavarot et al. 2003; Hua and Lappin 1995; Noble and Peacock 1996).

To corroborate the observed site-specific enantiomer selection in the ligated PpcA-Ru constructs, we also examined the unreacted Ru(bpy)_2_(Br-bpy) recovered from the ligation reaction mixtures for enantiomer content. The PpcA-Ru ligation reaction mixtures contained an estimated twofold molar excess of Ru(bpy)_2_(Br-bpy) relative to the PpcA proteins with single-site cysteine mutations. The near-UV CD spectra of the unreacted Ru(bpy)_2_(Br-bpy) fraction was obtained following the size-exclusion chromatographic separation of the PpcA-Ru constructs after completion of the coupling reaction. For each of the reaction mixtures, the unreacted Ru(bpy)_2_(Br-bpy) was found to be enriched in the enantiomer opposite to that of corresponding PpcA-Ru construct, and with optical activities that scale with the enantioselectivity of the construct, Fig. 4. These results demonstrate that site-specific enantiomer selective ligation is the cause for the near-UV CD optical activity for the PpcA-Ru constructs.

**Fig. 5:**
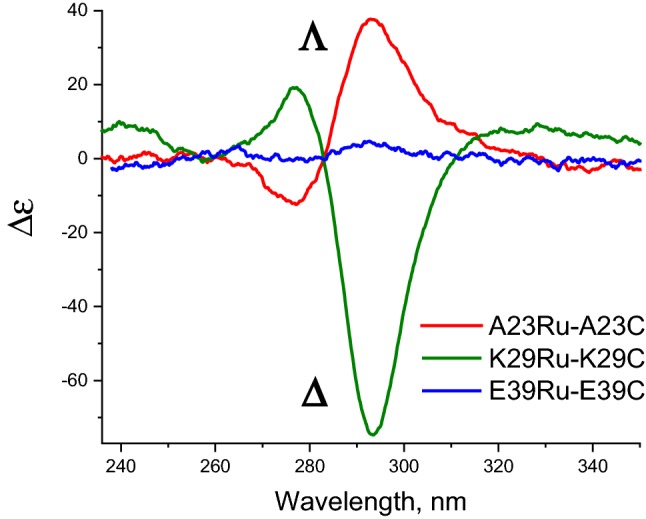
Near-UV CD difference spectra between [Ru(bpy)_3_]^2+^-linked constructs A23C-Ru, K29C-Ru, E39C-Ru and corresponding cysteine mutants, demonstrating the selective conjugation of Λ and Δ enantiomers. Experimental conditions: concentration of cytochrome 18.6–32.8 uM, pathlength 0.5 cm, 10 mM Tris buffer, 100 mM NaCl. The spectra presented as molar circular dichroism Δε, in M^−1^ cm^−1^. See text for normalization of spectra before subtraction of parental mutant spectra

In addition to the distinction in orientation, the magnitude of the Cotton effect is seen to differ among the three investigated constructs, Fig. 4. K29C-Ru shows the highest amplitude, A23C-Ru has intermediate, and for E39C-Ru the band was broad and barely detectable. Since the difference of the amount of linked photosensitizer might be contributing factor to this variance, before the quantitative comparison of optical activity we determined the fraction of conjugate in total protein sample applied for CD analysis. At first, analyses of metal atom content, Ru and Fe, for the PpcA-Ru constructs performed by ICP-MS. The results (Table S1, in Supporting information) show that in the samples used for near-UV CD investigations, the ratio of Ru to PpcA was 0.82 for A23C-Ru, 0.93 for K29C-Ru and 0.91 for E39C-Ru. These data were used to normalize the near-UV CD spectra and eliminate the underlying contributions from the PpcA host protein. Second, the parental mutant spectra were subtracted from the corresponding PpcA-Ru conjugates. The resulting normalized difference spectra, Fig. 5, illustrate more clearly the bisignate couplets consistent with *Λ* and *Δ* stereoisomers of [Ru(bpy)_3_]^2+^ (Browne et al. 2003; Caspar et al. 2003; Hua and Lappin 1995; Noble and Peacock 1996). They also allow a quantitative comparison of the magnitude of a Cotton effect for the three PpcA-Ru constructs.

From the normalized experimental optical activities, Fig. 5, the optical purity and enantiomeric excess can be calculated according to Eq. 5 (Schurig 2013) and using [*α*_max_] from the optical activity of a reference compound, cis-Λ-[Ru(bpy)_2_(py)_2_]Cl_2_ (Hua and Lappin 1995). From this, the fraction contribution of the Λ and Δ enantiomers and the stereoselective ratio, Δ/Λ, were tabulated for each of the PpcA-Ru constructs following the procedures outlined in Eqs. 6 and 7 (Carey and Sundberg 2007; Schurig 2013). The results are listed in Table 2. The K29C-Ru construct shows the highest enantiomer selectivity, with a preference for binding of Δ enantiomer with an enantiomer excess of 65.6%, corresponding to an enantiomeric proportion of approximately 5:1. The A23C-Ru shows a 33.1% enantiomer excess for the opposite, Λ enantiomer, with a Δ:Λ ratio of approximately 1:2. The E39C-Ru construct shows only a weak selectivity for the Λ enantiomer, with a measured excess of 4.2%. The differences in enantiomer selectivity for the each of photosensitizer labeling sites suggest that the details of the atomic structure configuration at the location of linkage are the major discriminating factors and that the magnitude of the selection is a measure of the relative stabilization energy for the two enantiomers.

**Table 2:** Optical activity and calculated enantiomeric proportions of conjugates

Conjugate	Experimental Δε at 295 nm [α]	Optical purity [α]/[α_max_]^a^	Enantiomeric excess %	Fraction Λ	Fraction Δ	Enantiomeric ratio E_major_/E_minor_	Enantiomeric selection ratio Δ/Λ
A23C-Ru	+ 37.73	0.331	33.1	0.665	0.335	2.003	0.504
K29C-Ru	− 74.73	0.656	65.6	0.172	0.828	5.105	5.105
E39C-Ru	+ 4.75	0.042	4.2	0.521	0.479	1.008	0.919

^a^[α_max_] = Δε_295_ = + 114 M^−1^ cm^−1^

### CD in the near-UV–Visible region (350–460 nm), heme cofactors

**Fig. 6:**
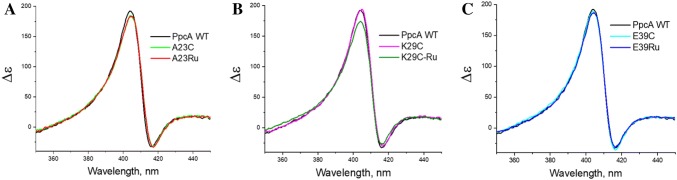
CD in the near-UV–Visible region, Soret band of hemes in cysteine mutants and constructs. a A23C-Ru; b K29C-Ru; c E39C-Ru. Experimental conditions: estimated concentration of cytochrome 20.6–32.8 uM, pathlength 0.5 cm, 10 mM Tris buffer, 100 mM NaCl. The spectra presented as molar circular dichroism Δε, in M^−1^ cm^−1^

The most prominent feature in the CD spectra associated with the optical transitions of the heme cofactors in the PpcA is the large positive CD peak located in the heme Soret absorption region, Fig. 6, similar to what has been reported for other hemeproteins. The optical activity of heme groups in near-UV (L band) and visual regions (B band region) of the absorption spectrum are understood to arise from the acquired chirality of the heme integrated with the asymmetrical protein environment (Blauer et al. 1993; Schweitzer-Stenner 2011; Woody and Pescitelli 2014). The rotational strength of heme optical transitions is generally determined by the Coulomb interactions between the heme side chains and the protein backbone in its binding pocket (Hsu and Woody 1971). The vinyl group torsions were considered to be the major determinant of the intensity and orientation of CD signal in hemoglobin and myoglobin (Woody and Pescitelli 2014), but the propionate group orientations and interactions also make significant contributions into the appearance of the CD spectrum (Nagai et al. 2015, 2018).

In c-type cytochromes, the vinyl groups of the hemes are involved in the thioether linkage to the polypeptide chain, where they aligned very specifically. Namely, the 3-vinyl group is always attached to the N-terminal cysteine and the 8-vinyl group to the C-terminal cysteine of the CXXCH binding motif. As a result, the same orientation of heme with respect to the attachment sequence is found in all structures obtained so far (Barker and Ferguson 1999). Thus, the similar contributions to the CD spectrum are expected from these side chains. In contrast, the alignments of propionate groups are very diverse among c-heme structures, resulting in the variations of CD spectra for this type of cytochromes. In addition, further structural features of heme binding in cytochromes c, considered to be negligible for myoglobin and hemoglobin, such as porphyrin macrocycle deformations in binding pocket, the π–π* transitions of the histidine ligand, and the polarizability of thioether bonds in the close proximity to the heme, are also known to induce the optical activity in c-type cytochromes (Blauer et al. 1993; Schweitzer-Stenner 2011). All these factors bring the rotational strength of different direction and intensity in the B band, which, as a result, is split into two components of opposite sign (Schweitzer-Stenner 2008). This type of splitting is apparent in the multi-heme cytochrome PpcA also, and, in addition to the positive Cotton effect with the maximum at 404 nm, the native protein and constructs display some negative trough with the minimum at 417 nm.

The introduction of a cysteine mutation decreased the amplitude of the maximum response in A23C by 3.5% as compared to wild-type PpcA, and even less in E39C mutant, but it did not alter the heme optical activity in K29C variant. Concurrently, the whole doublet slightly shifted to the lower energy in A23C as well as the maximum in K29C. While CD spectra of constructs qualitatively reproduce the position and the magnitude of maxima and minima for mutants, some differences are evident. In A23C-Ru, there is no amplitude change compared to A23C, and doublet was shifted slightly to the lower energy relative to native cytochrome, similar to parent mutant. In K29Ru construct there was the 7.7% decrease in amplitude relative to K29C, but the position of the CD maximum was equivalent to that in the wild-type cytochrome. For E39C and E39C-Ru a progressive decrease in the CD amplitude was seen. These spectral differences might arise from variations in heme–protein or heme–heme excitonic coupling due to changes in electrostatic interactions at the place of mutation. At the same time, the covalent linkage of the [Ru(bpy)_3_]^2+^ molecule in PpcA-Ru constructs can also contribute to the detected small perturbation in the CD spectra.

### Molecular dynamics simulations

To evaluate and visualize dynamics of the PpcA-Ru constructs, we performed triplicate 300–400 ns MD simulations for each of the A23C-Ru, K29C-Ru, and E39C-Ru constructs. Within these trajectories the distances of closest approach between conjugated atoms on the bipyridyl ligands of the linked [Ru(bpy)_3_]^2+^ and non-hydrogen atoms of PpcA and heme cofactors (see Fig. S2 and S3 for assignment of atoms) were tracked to provide a measure of conformational flexibility within thermally equilibrated conformers (Fig. S4-6, S9 in Supplemental Information). For the K29C-Ru construct simulations were performed using the predominant Δ-[Ru(bpy)_3_]^2+^ enantiomer. Since the enantiomer preference was less pronounced for A23C-Ru, with the enantiomer ratio of 1:2 in favor of Λ enantiomer and approximately a third of population carrying the Δ form, we performed MD simulations for both Λ and Δ enantiomers of [Ru(bpy)_3_]^2+^. MD simulations were carried out for Λ-E39C-Ru conjugate for comparison to a site which displayed negligible enantiomer selectivity.

**Fig. 7:**
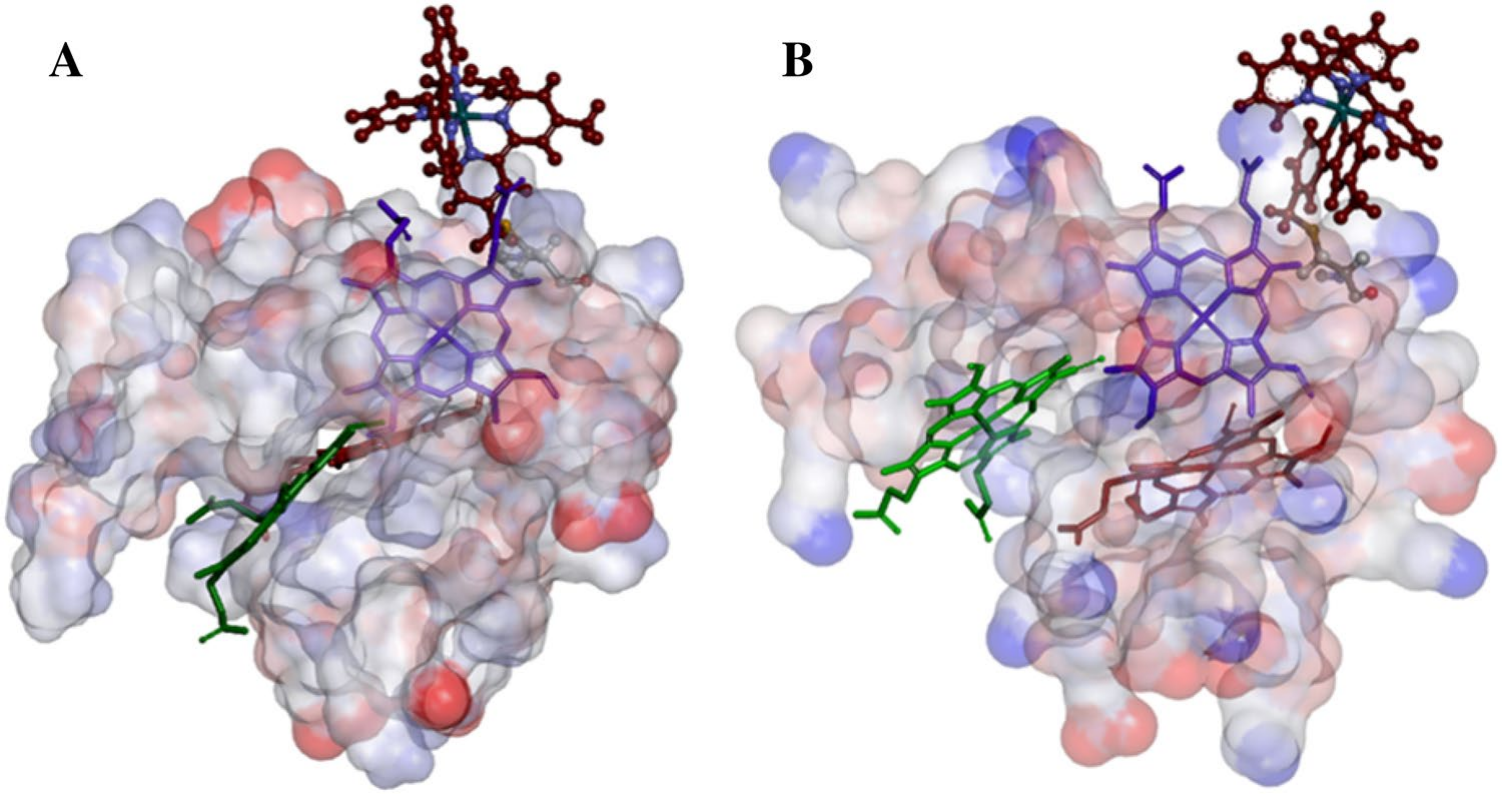
Comparison of two representative equilibrium conformations for Λ-[Ru(bpy)_3_]^2+^ in the A23C-Ru construct modeled by MD simulation. The colors represent the surface charge distribution of A23C molecule, ranging from negative (red) to positive (blue). a The ligated [Ru(bpy)_3_]^2+^ wedged between Heme III propionates; b A conformer in which the ligated Λ-[Ru(bpy)_3_]^2+^ is rotated around one of the propionate chains. This propionate sits in the groove between two bpy ligands

The examination of fluctuations in the distances of closest approach between [Ru(bpy)_3_]^2+^ ligand carbon atoms and those of the closest non-hydrogen atoms of protein and heme reveal differences in the MD trajectories for the two enantiomers of A23C-Ru construct. The frequency of oscillations in Λ is seen to be much lower compared to the Δ enantiomer for the A23C-Ru construct (Fig S4 and S5). Larger amplitudes of motion occurring in shorter times in the Δ enantiomer are indications of much higher [Ru(bpy)_3_]^2+^ velocities and more significant molecular forces acting from the protein toward the photosensitizer moiety. This, in turn, suggests that we are trying to insert a Δ enantiomer molecule into the space which does not have an optimal complementary shape. Even for the preferred Λ-A23C-Ru construct, the distance fluctuations were found to be far more significant compared to those for K29C-Ru (Fig. S6). In addition, the two clear subsets of structures from one ~ 140 ns trajectory of Λ-A23C-Ru indicated the presence of the two conformations upon binding (Fig. 7). In one of them the [Ru(bpy)_3_]^2+^ molecule is wedged between heme III propionates, in the second it rotates around one of the propionates which then gets in the groove between two bpy ligands (Fig. S7 illustrates the close-up look at the position of photosensitizer relative to heme III).

MD simulations for the A23C-Ru and K29C-Ru conjugates found that among carbon atoms involved in the covalent attachment of [Ru(bpy)_3_]^2+^ to the engineered cysteine residues, C4A-CAM-SG-CB-CA-protein backbone (see Figure S2 for assignment of atoms), the CAM carbon of methylene bridge has the most rigid position. This atom is situated between the nitrogen of pyridine ring and sulfur of thioether bond and was located within 4–5 Å from heme III in both, A23C-Ru and K29C-Ru, and in all simulations performed. Further, in the A23C-Ru conjugate, it is the closest to heme III of all atoms. In K29C-Ru more atoms, namely C4A–C6A of adjoining pyridine ring, can be detected in the direct vicinity to the heme III aromatic groups. The distances between these atoms and protein do not change much (less than 1–2 Å) during 400 ns of all three simulations performed. For K29C-Ru equilibration with Δ enantiomer took about 50 ns, after which all distances were almost stable suggesting only one conformation of [Ru(bpy)_3_]^2+^. When increased distances were detected during short stretches of time, they were concurrent and reversible and possibly indicated the conformational changes in the atom positions for the amino acid residues closest to [Ru(bpy)_3_]^2+^. In the case of a movement of [Ru(bpy)_3_]^2+^ with respect to a fixed protein site, the simultaneous shortening of some distances along with elongation of others would be apparent rather than increases across the board observed here.

**Fig. 8:**
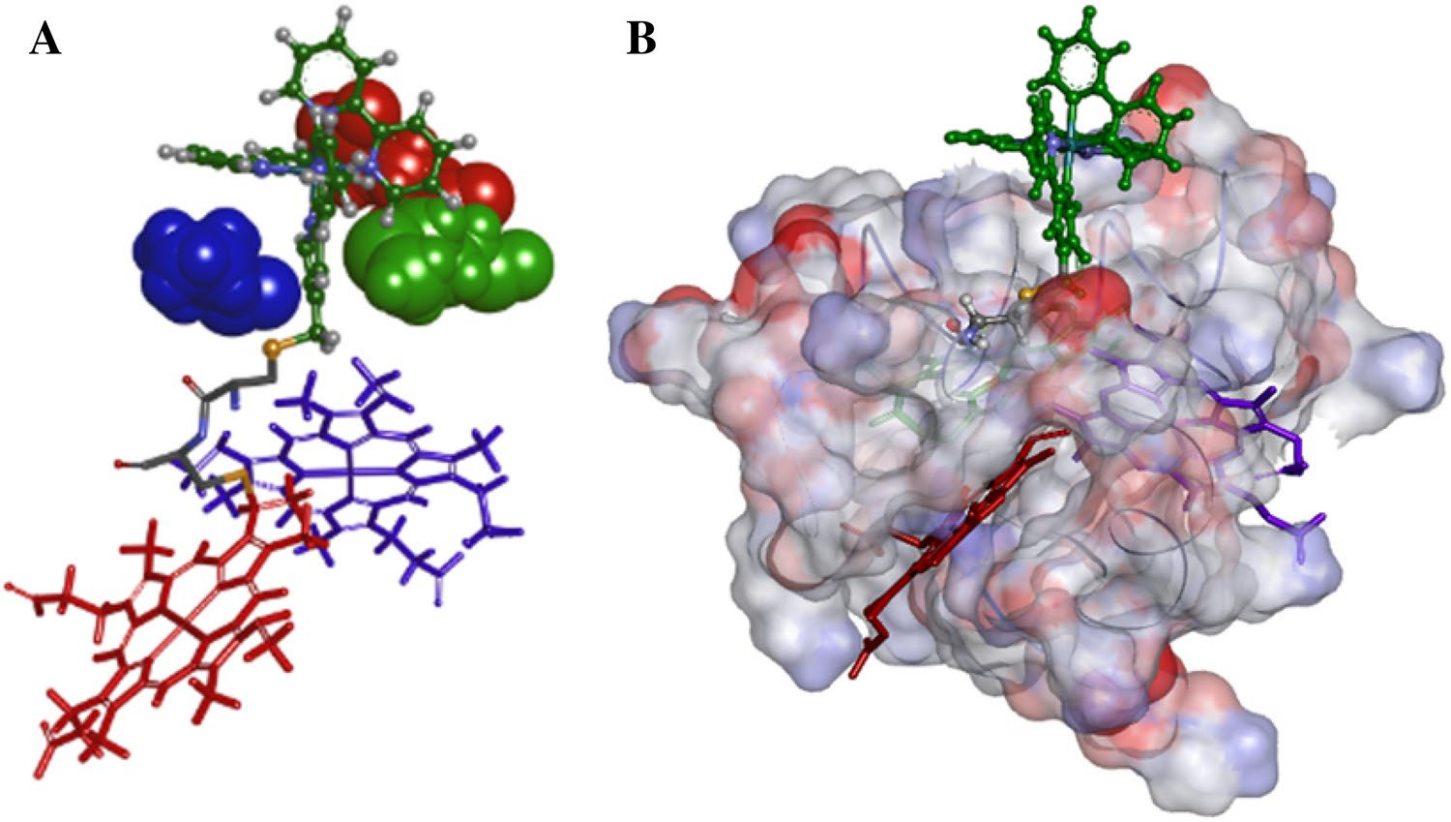
An equilibrium conformer for the Δ-K29C-Ru conjugate according to MD simulations. a The cofactor arrangement and positions of amino acid side chains confining the Δ enantiomer of the [Ru(bpy)_3_]^2+^ chromophore relative to heme I (red) and heme III (violet), illustrating the spatial constraints formed by surrounding amino acids: Lys49 (blue), Glu57 (red) and Met58 (green); b Protein surface charge presentation of the positioning the conjugated [Ru(bpy)_3_]^2+^ molecule on the surface of protein scaffold illustrating its steric stabilization and disposition relative to hemes

A closer look at the Δ-K29C-Ru structures shows that CAM carbon forms a contact point with one of the heme III vinyl carbons, namely 3-vinyl group (Fig. S8). We also observed Δ-[Ru(bpy)_3_]^2+^ molecule becoming lodged between side chains of Lys49, Cys54, Glu57 and Met58, resulting in the steric stabilization in the vicinity of this heme (Fig. 8). This is an indication of one preferable conformation of photosensitizer and the modeling is also consistent with CD data—if there were enough space for [Ru(bpy)_3_]^2+^ movement or several distinct conformations, we would not have observed a strong preference for one enantiomer over the other.

Within these MD simulations, conformational flexibilities of the PpcA-Ru constructs with respect to the heme cofactor were also tracked specifically by examining the distances of closest approach between aromatic ligand atoms of the [Ru(bpy)_3_]^2+^ photosensitizer and aromatic atoms of the closest PpcA heme cofactor in the Λ-A23C-Ru, Δ-K29C-Ru, and Λ-E39C-Ru conjugates. Since the proximity of redox centers is a key factor determining donor–acceptor orbital overlap in biological electron transfer (Beratan et al. 1992; Page et al. 1999), the tracking of minimum distances between aromatic atoms of donor–acceptor pairs provides an indication of relative scale for electron transfer during the simulated trajectories, and provides a measure of the conformational flexibility for the photosensitizer molecule in each of the constructs. For constructs modeled with the preferred enantiomer, the Δ for K29C-Ru or Λ for A23C-Ru and E39C-Ru, a low variation in distances (left panels, Fig. S9 in Supplemental Information) was found to be correlated with the higher enantiomer selectivity. For A23C-Ru the mean minimal distances between aromatic atoms were determined as 6.03 ± 0.77 Å, 6.31 ± 0.88 Å, and 5.98 ± 0.83 Å, respectively for the 3 independent MD simulations. For K29C-Ru the mean minimum distances were 6.87 ± 0.51 Å, 6.98 ± 0.58 Å, 6.98 ± 0.57 Å for the three simulations. The lower standard deviation of this parameter in K29C-Ru compared to the A23C-Ru construct points out to the more restricted mobility for the K29C-Ru versus A23C-Ru linking sites and correlates with the higher enantiomer selectivity observed for the K29C-Ru compared to A23C-Ru construct. In contrast, the widest distributions of the minimal distances were observed in E39C-Ru simulations, with mean minimal distances of 11.94 ± 2.01 Å, 12.84 ± 1.77 Å, 13.26 ± 1.66 Å in three independent simulations, corresponding to the most flexible, among the three conjugates, position of [Ru(bpy)_3_]^2+^. This flexibility corroborates the low, almost absent selectivity, in enantiomer binding for this construct.

## Discussion

The results of our experiments demonstrate the chiral discrimination exhibited by the environment of the cysteine residue tethering the [Ru(bpy)_3_]^2+^ molecule, which governs the stereochemical selection in the conjugation of the photosensitive group to protein matrix. All three sites of cysteine mutations purposely were introduced in distinctive parts of the PpcA polypeptide chain, thus, they and the extrinsic groups bound to them were anticipated to experience different interactions with the protein matrix. The constructs indeed had diverse characteristics, in particular, excited-state PET times, for the K29C-Ru, A23C-Ru, and E39C-Ru conjugates were 6 ps, 130 ps, and 35 ns and correlated with distances between adjoined photosensitizer moiety and closest heme cofactor (Kokhan et al. 2015).

The CD analysis demonstrated mostly small modifications in the secondary structure of protein backbone for the slowest of constructs, E39C-Ru, with only about 4% of ee for Λ enantiomer. In the crystal structure of PpcA Glu39 residue is located in a one-turn helix within a flexible region of the polypeptide chain, forming a large solvent-accessible opening (Pokkuluri et al. 2004). This fragment has just a few contacts with the rest of the protein and a molecule of deoxycholic acid is filling in this area, stabilizing the loop, which may otherwise have multiple conformations. With the lack of fixed geometry, it was unlikely that at this position accessory molecule will produce steric clashes with atoms of the protein core. This determines its acceptability for conjugation reaction and relatively low stereoselectivity, as established in the CD experiments. Attachment of the Ru(bpy)_2_(Br-bpy) in this flexible segment places it close to the surface of the protein molecule and makes it exposed to the solvent. In addition, such arrangement predetermined the much longer, relative to other constructs, distances between the photosensitizer molecule and heme cofactors and resulted in lower PET rates.

As evidenced by the crystal and solution structures (Morgado et al. 2012), Ala in position 23 is located in a flexible two-turn α-helix region and within van der Waals interaction distances with the propionate side chain attached to C-13 of heme III (see Figure S3 for assignment of heme atoms). Because of the volume of the added photosensitizer molecule in A23C-Ru construct, such proximate arrangement to heme could create close contact, even steric constraints for linked [Ru(bpy)_3_]^2+^. Filling up the space intended for a much smaller amino acid residue with a cysteine-tethered [Ru(bpy)_3_]^2+^ would require very precise positioning of the inserted photosensitizer. Such spatial restrictions create the conditions for stereoselectivity, detected in our experiment preferential binding of Λ enantiomer in this case. In addition, the attachment of the [Ru(bpy)_3_]^2+^ molecule led to a significant change of the net charge in the area surrounding the binding site, as a consequence of gaining the positively charged compound in the place of small hydrophobic Ala. Furthermore, having the net +2 charge, [Ru(bpy)_3_]^2+^ is expected to interact with closest negatively charged propionate of heme III, but due to the added volume, it might be in the vicinity of both propionate groups. By acting through steric hindrance rather than ionic attraction or repulsion this interference would also affect the positioning of added chemical group. By means of MD simulations, we were able to make a more detailed analysis of probable interference of the linked [Ru(bpy)_3_]^2+^ with heme cofactors and contribute to the visualization of its positioning with respect to the PpcA protein matrix. At the same time, the further corroboration would be advantageous for the inquiry about the extent of each enantiomer interaction with the heme cofactor and protein matrix in A23C-Ru.

The Lys residue in position 29, the second of two in the CKKCH binding motif of heme I, is situated within the 3_10_-helix forming its attachment site in the native PpcA structure. However, the side chain of this amino acid is turned in the direction of vinyl groups anchoring heme III, making it adjacent to both hemes. Substitution of this bulky charged amino acid by Cys vacated some space in this unique arrangement along with the loss of positive charge. Binding of Ru[(bpy)_3_]^2+^ photosensitizer qualitatively, or at least to some extent, restored the charge distribution existed before the introduction of mutation, probably concurrently increasing the extent of β structures at the expense of α helices. But due to the bigger volume and different shape, a new molecule had significant spatial constraints while entering the existing cavity, the shape of which allowed accommodation of Δ enantiomer with a higher level of specificity. The selection of this enantiomer in a binding event occurs because it better complements the geometry of protein matrix and this drives the equilibrium to its preferential conjugation. The close-fitting, almost immobilization of artificial photosensitive group in the vicinity of hemes ensured the immediate interactions and became prerequisite for ultrafast PET. The additional aspect was its positioning in the area of conjugated vinyl groups, themselves having a conserved arrangement in the covalently bound heme c cofactors. Such location, in combination with higher stereoselectivity, contributes to the faster rates in K29C-Ru compared to A23C-Ru. Along with the shorter through-peptide pathway to heme I, the through-space pathway to heme III involves the conjugated vinyl group in K29C-Ru compared to the saturated bonds of propionates for A23C-Ru.

Thus, the degree of site-specific stereoselection in the conjugation of [Ru(bpy)_3_]^2+^ can be an indicator for the precision of docking photosensitizer geometry and interaction at a linking site, the complementary shape and restriction of mobility of adjoined photosensitizer. Because the conformational specificity is required to achieve the controlled PET by design, the opportunity exists to exploit stereoselection as the way to achieve specific, structured cofactor pair geometries for optimized PET. The ultrafast PET rates observed for the K29C-Ru and E23C-Ru constructs (Kokhan et al. 2015) implies the a close proximal positioning between the conjugated photosensitizer and heme cofactors, a condition where stereo-specific structural differences between the [Ru(bpy)_3_]^2+^ enantiomers could be expected to emerge as a feature of the site-specific Ru[(bpy)_3_]^2+^ interaction. For the flexible loop region encompassing the E39C-Ru site, we would anticipate a less rigorous enantiomer selectivity. The results presented here on the chiral selectivity for each of these sites demonstrate the validity of these expectations and offer strategies for moving forward to exploit chiral selectivity as a means to design refined tuning of PET conjugates in manner that mimics the site-specific tuning of cofactors in redox protein electron transfer and photosynthesis.

## Electronic supplementary material

Below is the link to the electronic supplementary material.

Supplementary material 1 (DOCX 3085 kb)
