# Supplementary material for: Examination of abiotic cofactor assembly in photosynthetic biomimetics: site-specific stereoselectivity in the conjugation of a ruthenium(II) tris(bipyridine) photosensitizer to a multi-heme protein
Source: Photosynth Res. 2020 Jan 10;143(2):99–113. doi: 10.1007/s11120-019-00697-8 (PMC6989566; doi:10.1007/s11120-019-00697-8)
Supplement: Supplementary file 1 — Supplementary material 1 (DOCX 3085 kb) [file 11120_2019_697_MOESM1_ESM.docx]

**Photosynthesis Research**

**Supplemental Information**

**Examination of Abiotic Cofactor Assembly in Photosynthetic Biomimetics – Site-Specific Stereoselectivity in the Conjugation of a Ruthenium(II) Tris(Bipyridine) Photosensitizer to a Multi-Heme Protein**

Nina S. Ponomarenko,^1,^* Oleksandr Kokhan^3^, Phani R. Pokkuluri^2^, Karen L. Mulfort^1^, and David M. Tiede^1,^*

^1^Chemical Sciences and Engineering Division and ^2^Biosciences Division, Argonne National Laboratory, 9700 South Cass Avenue, Argonne, Illinois 60439, United States

^3^ Department of Chemistry and Biochemistry, James Madison University, 901 Carrier Drive, Harrisonburg, VA 22807

Corresponding authors:

*David M. Tiede [tiede@anl.gov](mailto:tiede@anl.gov)

*Nina S. Ponomarenko [ponomarenko@anl.gov](mailto:ponomarenko@anl.gov)

**Table S1** Percentage content of bound [Ru(bpy)_3_]^2+^ PpcA in samples used for CD determined by ICP measurements

| Sample | Absorption  Ratio 286/350 nm | Fe  content  uM | PpcA  concentration  uM | Ru  content  uM | Ratio  Ru to PpcA | [Ru(bpy)_3_]^2+^ bound to PpcA, % |
| --- | --- | --- | --- | --- | --- | --- |
| PpcA wild type | 0.29 | 14.907 | 4.969 | 0.015 | 0.0030 | 0.30 |
| A23C | 0.37 | 16.929 | 5.643 | 0.024 | 0.00431 | 0.43 |
| A23C-Ru | 1.12 | 15.658 | 5.219 | 4.267 | 0.818 | 81.8 |
| K29C | 0.41 | 17.302 | 5.767 | 0.017 | 0.00290 | 0.29 |
| K29C-Ru | 1.16 | 14.872 | 4.957 | 4.623 | 0.933 | 93.3 |
| E39C | 0.36 | 16.857 | 5.619 | 0.017 | 0.00339 | 0.34 |
| E39C-Ru | 1.10 | 15.892 | 5.297 | 4.825 | 0.9109 | 91.1 |


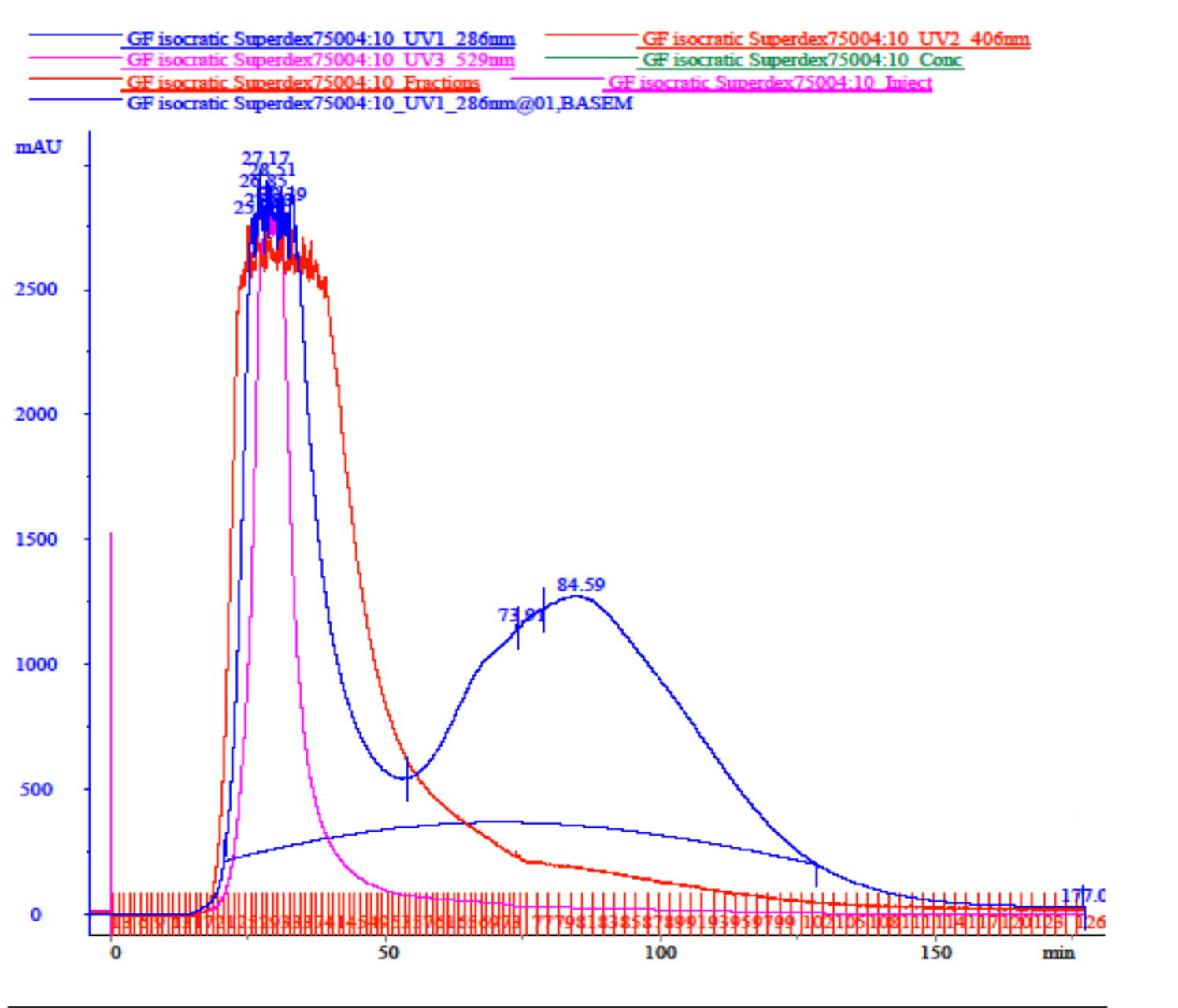


**Fig.S1** Isoctatic gel filtration chromatography of K29C-Ru after binding reaction illustrating separation of Ru(bpy)_3_-conjugated cytochrome from non-reacted Ru(bpy)_3_-Br. Elution profile monitored at following wavelength – blue line 286 nm – peak of Ru(bpy)_3_-Br absorption; magenta 529 nm – peak of PpcA absorption in Q band of the visible spectral region; red 406 nm - peak of PpcA absorption in Soret band.


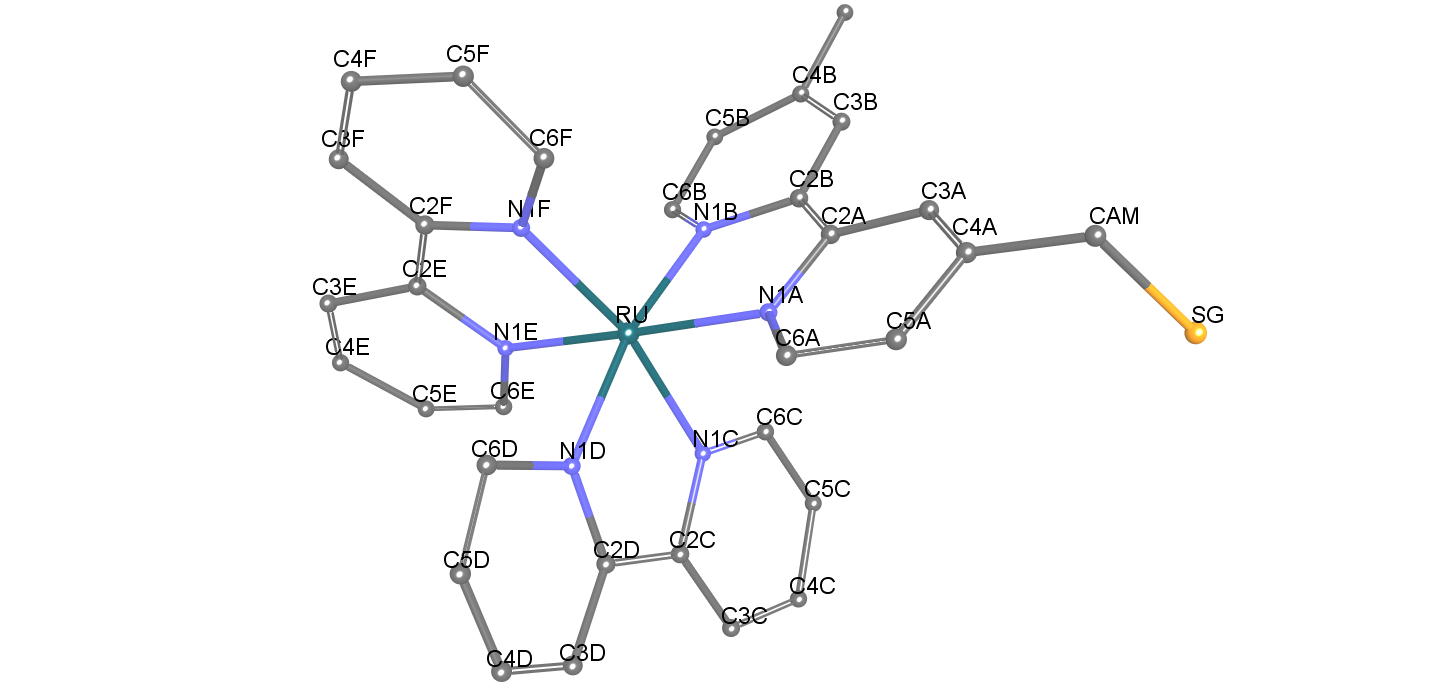


**Fig.S2** Molecular structure of [Ru(bpy)_3_]^2+^ with the atom numbering system. Hydrogen atoms are omitted for clarity. Molecule is shown with bond to PpcA mutant through sulfur of cysteine, based on model 1 of Λ Ru(bpy)_3_^2+^ in A23Ru.


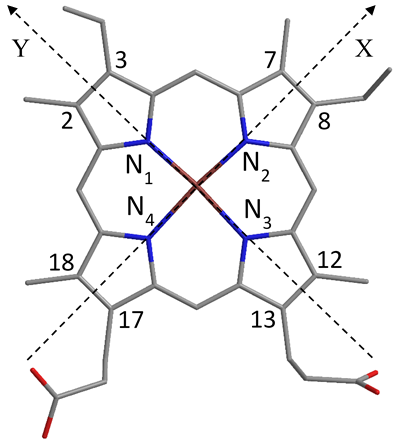


**Fig.S****3** Diagram of heme c showing the IUPAC numbering of atoms. When one looks down at the heme from the sixth coordination site of the heme c, the substituents are arranged in a clockwise order with increasing numbers: four methylene groups at position 2,7,12,18; two vinyl groups at positions 3 and 8; propionate groups at 13,17.


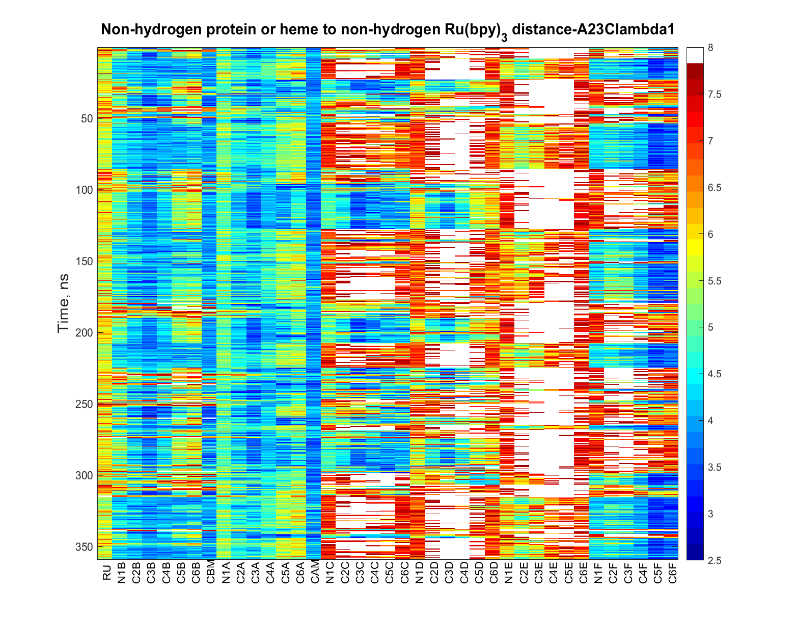


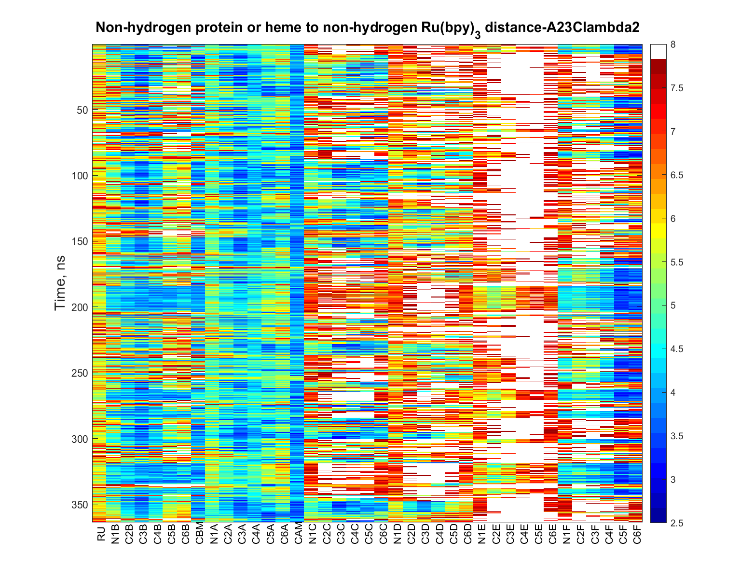


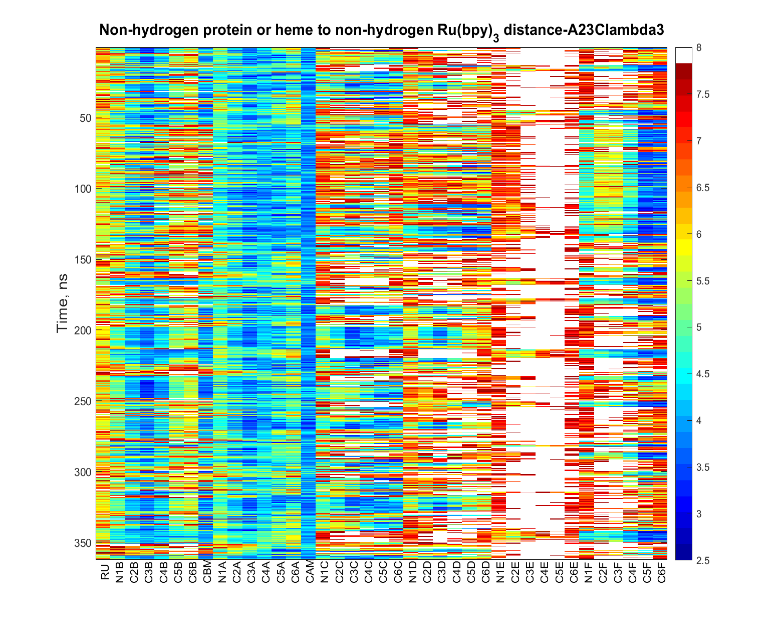


**Fig.S4** MD simulations for A23C-Ru Λ enantiomer. The closest distances in Å for each non-hydrogen atom of [Ru(bpy)_3_]^2+^ (X-axis) to non-hydrogen atoms of PpcA excluding only the attachment residue Cys23.


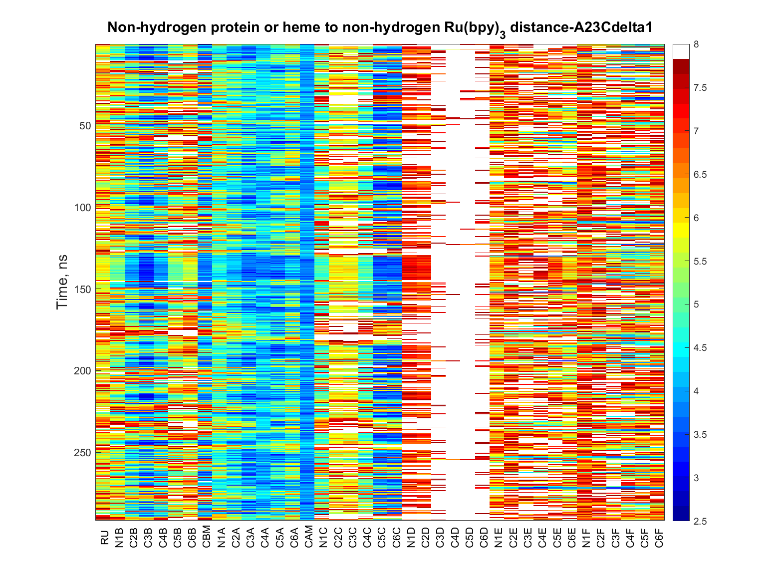


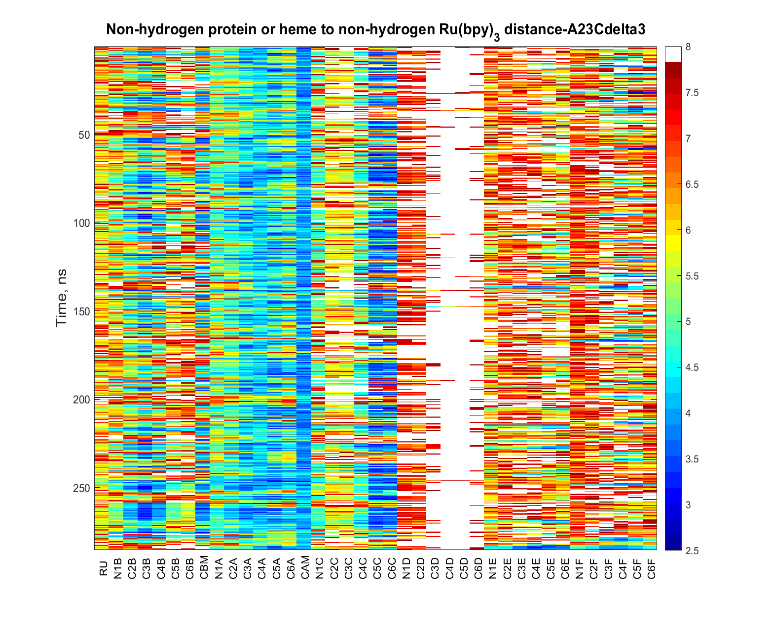


**Fig.S5** MD simulations for A23C-Ru Δ enantiomer. The closest distances in Å for each non-hydrogen atom of [Ru(bpy)_3_]^2+^ (X-axis) to non-hydrogen atoms of PpcA excluding only the attachment residue Cys-23.


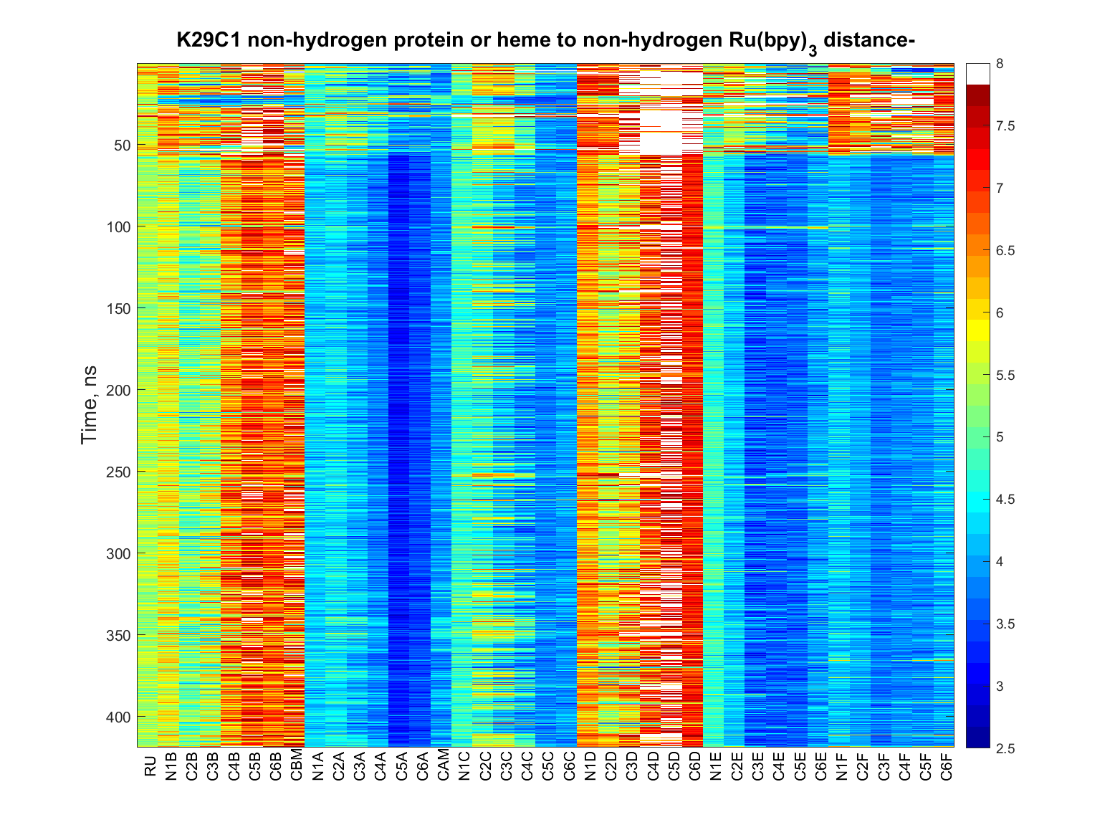

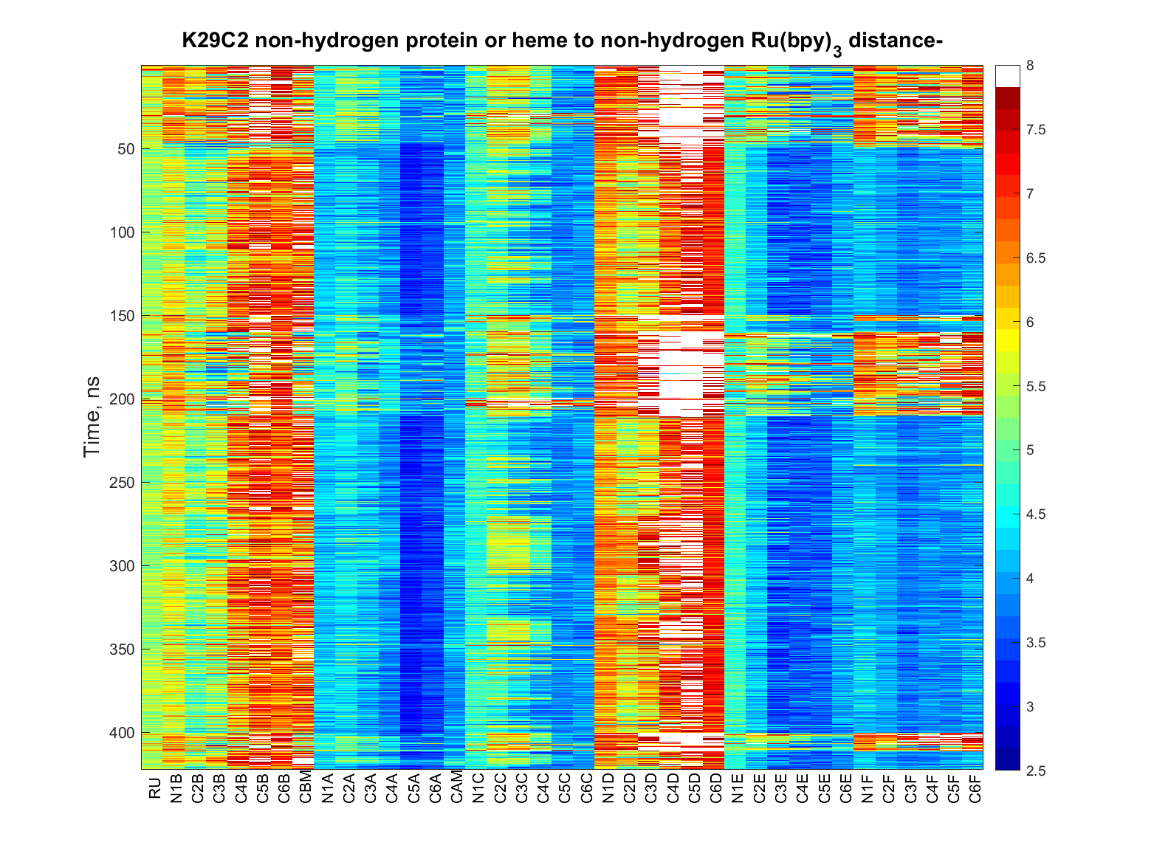

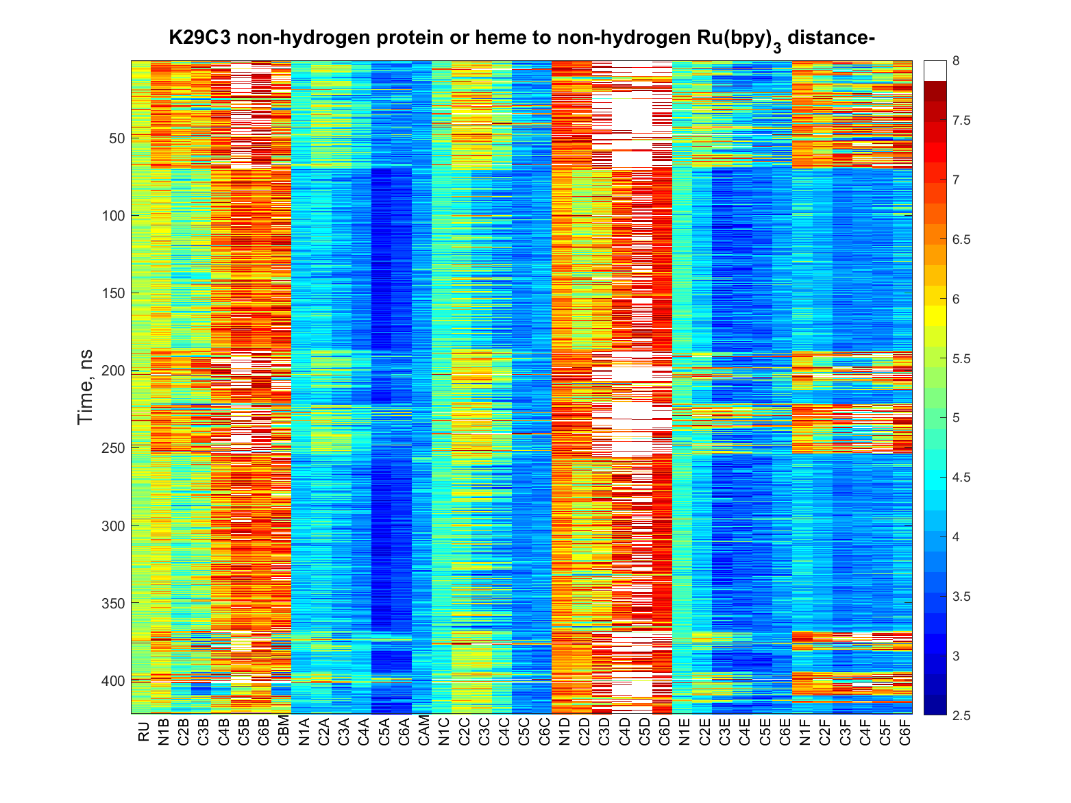


**Fig.S6** MD simulations for K29C-Ru Δ enantiomer. The closest distances in Å for each non-hydrogen atom of [Ru(bpy)_3_]^2+^ (X-axis) to non-hydrogen atoms of PpcA excluding only the attachment residue Cys-29 during 450 ns equilibration.

On the first panel - equilibration took about 50 ns after which all distances were stable suggesting only one conformation of [Ru(bpy)_3_]^2+^. In contrast, for the other two panels short stretches of time are detected with the increased distances. This possibly means the conformational changes in the closest to [Ru(bpy)_3_]^2+^ amino acid residues, as in case of a different conformation of [Ru(bpy)_3_]^2+^ the shorter distances somewhere else at the same time would be apparent, rather than across the board increases observed here.


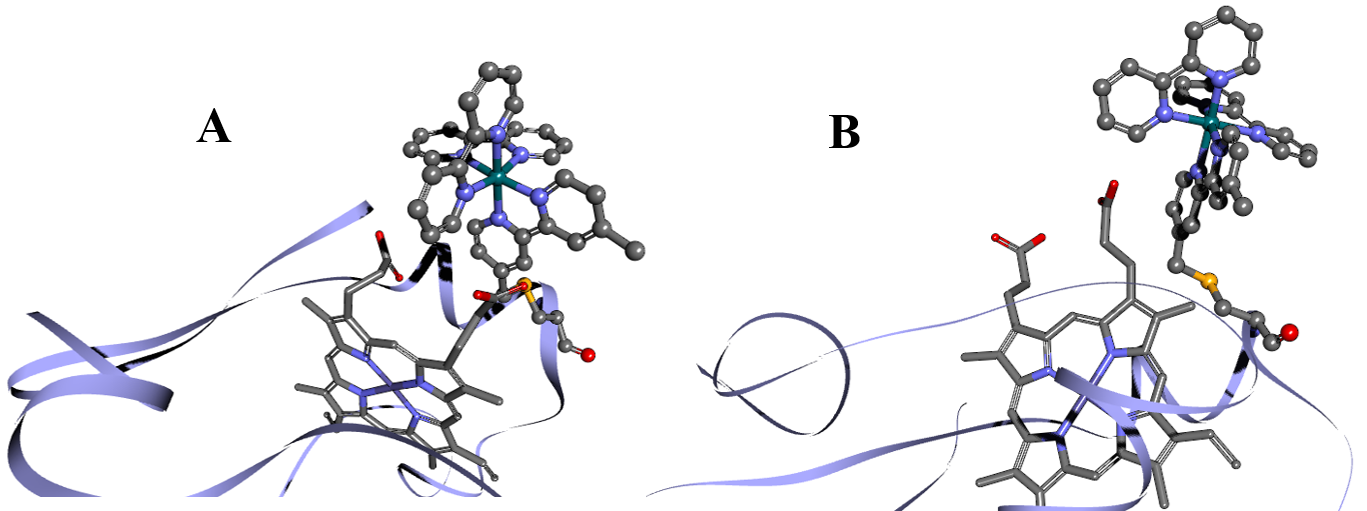


**Fig. S7.** The close-up look at two representative equilibrium conformations for Λ-[Ru(bpy)_3_]^2+^in the A23C-Ru construct modeled by MD simulation. **A.** [Ru(bpy)_3_]^2+^ wedged between Heme III propionates; **B.** A conformer in which the ligated Λ-[Ru(bpy)_3_]^2+^ is rotated around one of the propionates and sits in the groove between two bpy ligands.


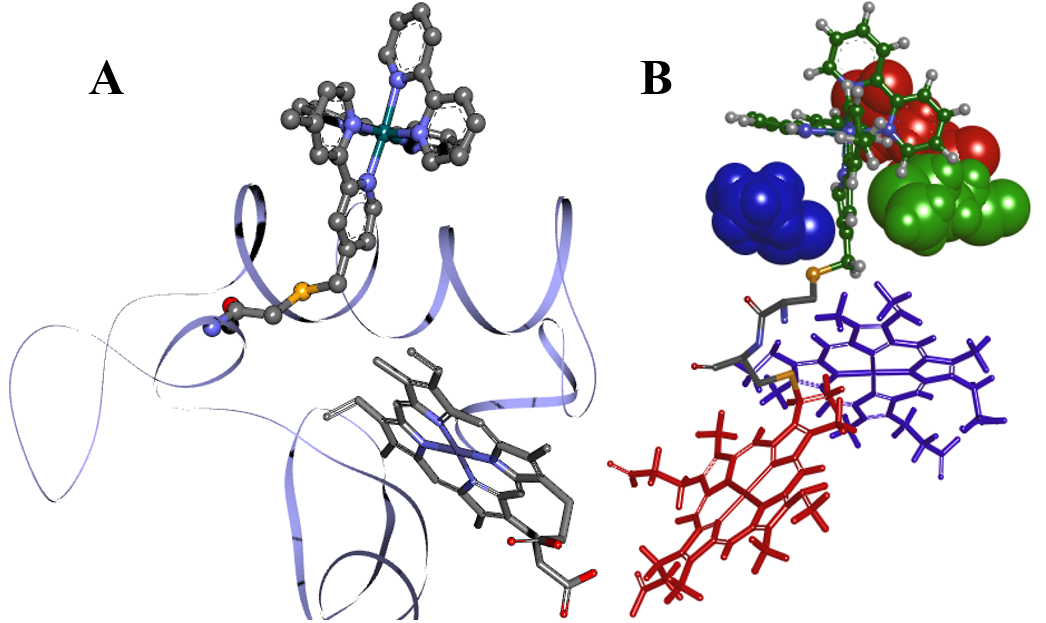


**Fig. S8.** The close-up look at model of Δ-K29C-Ru. The arrangement of [Ru(bpy)_3_]^2+^ chromophore relative to heme III and polypeptide chain.

**Fig. S9.** Minimal distances between aromatic atoms of [Ru(bpy)_3_]^2+^ photosensitizer and aromatic atoms of the closest PpcA hemes in Λ-A23C-Ru, Δ-K29C-Ru, and Λ-E39C-Ru conjugates, modeled with its preferred enantiomer. Plots in the left column show minimal distances as a function of time for 3 independent MD simulations performed for each conjugate. Plots in the right column illustrate distributions of distances from the simulations of corresponding constructs.
